# Cuticular hydrocarbons promote desiccation resistance by preventing transpiration in *Drosophila melanogaster*

**DOI:** 10.1242/jeb.247752

**Published:** 2024-11-28

**Authors:** Kamar Nayal, Joshua J. Krupp, Osama H. M. H. Abdalla, Joel D. Levine

**Affiliations:** ^1^ Department of Biology, University of Toronto at Mississauga, 3359 Mississauga Road, Mississauga, ON, Canada, L5L 1C6; ^2^ Department of Ecology and Evolutionary Biology, University of Toronto, 25 Willcocks Street, Toronto, ON, Canada, M5S 3B2

**Keywords:** Oenocyte, Insects, Evaporative water loss, Water balance, Stress response

## Abstract

Desiccation is a fundamental challenge confronted by all terrestrial organisms, particularly insects. With a relatively small body size and large surface-to-volume ratio, insects are susceptible to rapid evaporative water loss and dehydration. To counter these physical constraints, insects have acquired specialized adaptations, including a hydrophobic cuticle that acts as a physical barrier to transpiration. We previously reported that genetic ablation of the oenocytes – specialized cells required to produce cuticular hydrocarbons (HCs) – significantly reduced survivorship under desiccative conditions in the fruit fly, *Drosophila melanogaster*. Although increased transpiration – resulting from the loss of the oenocytes and HCs – was hypothesized to be responsible for the decrease in desiccation survival, this possibility was not directly tested. Here, we investigated the underlying physiological mechanisms contributing to the reduced survival of oenocyte-less (oe−) flies. Using flow-through respirometry, we show that oe− flies, regardless of sex, exhibited an increased rate of transpiration relative to wild-type controls, and that coating oe− flies with fly-derived HC extract restored the rate to near-wild-type levels. Importantly, total body water stores, including metabolic water reserves, as well as dehydration tolerance, measured as the percentage of total body water lost at the time of death, were largely unchanged in oe− flies. Together, our results directly demonstrate the critically important role played by the oenocytes and cuticular HCs to promote desiccation resistance.

## Teaser

**Highlighted Article:** Cuticular hydrocarbons are demonstrated to be directly responsible for promoting desiccation resistance by reducing the rate of water loss via transpiration in *Drosophila melanogaster*.

## INTRODUCTION

Terrestrial animals are uniquely adapted to cope with adverse environmental conditions where desiccation is a fundamental threat to survival. This is certainly true of insects, which due to a relatively small body size and high surface-to-volume ratio are particularly susceptible to evaporative water loss and dehydration (Gibbs, 2002b; Gibbs and Rajpurohit, 2010). The ecological success of insects, which inhabit even the most arid environments, has been largely attributed to their considerable capacity to manage and conserve internal water stores (Beyenbach, 2016; Chown and Nicolson, 2004; Edney, 1977). Desiccation-resistant traits among insect species are diverse and varied, and have been linked to geographic location and environmental conditions (i.e. temperature and water availability; Thorat and Nath, 2018).

The insect integument, consisting of the cuticle and underlying epidermis, is vital to preventing transpiration – the predominant route of water loss under desiccative conditions. Transpiration has been estimated to account for approximately 70% of water loss to the external environment; respiration and excretions comprise the remainder (Quinlan and Gibbs, 2006; Wang et al., 2021). The epicuticle, a waxy layer coating the cuticular surface consisting primarily of a complex mix of lipids, is of particular importance (Edney, 1977; Hadley, 1989). Diffusion of water through the hydrophobic epicuticular lipids is the rate-limiting step in preventing transpiration (Gibbs, 2011). Many insect and arachnid species exhibit a marked increase in cuticular permeability when epicuticular lipids are chemically removed, mechanically disrupted, adsorbed by dust or altered by high temperatures (Gibbs, 2002a, 2011; Hadley, 1989).

The epicuticular lipids of insects consist primarily of hydrocarbons, the physical properties of which are determined by chain-length, saturation level and side-chain composition. These cuticular hydrocarbons (HCs) typically include variable quantities of straight-chain *n*-alkanes with lengths of 20–40 carbons (with odd-number carbon chains in the middle of this range often the most abundant), branched alkanes possessing one or more methyl groups (methyl-branched alkanes) and unsaturated alkenes possessing one or more *cis* double bonds [(*Z*)-alkenes and (*Z*,*Z*)-alkadienes] (Blomquist and Bagnères, 2010; Yew and Chung, 2015). Although representing a diverse set of compounds, each with different properties (melting temperature, *T*_m_, packing density, etc.), HCs are, in general, the most hydrophobic of the epicuticular lipids and are therefore thought to most directly affect cuticular permeability.

In *Drosophila*, many studies have linked HCs to desiccation resistance, defined, in part, by the ability to prevent the loss of internal water stores. Approaches utilizing artificial selection (Ferveur et al., 2018; Gibbs et al., 1997; Telonis-Scott et al., 2006), ecological comparisons (Gibbs and Matzkin, 2001; Rouault et al., 2004; Wang et al., 2022) and quantitative genetic analyses (Dembeck et al., 2015; Foley and Telonis-Scott, 2011) have concluded that while desiccation resistance involves multiple mechanisms, the composition of HCs is perhaps the most important determinant of cuticular permeability. Precise genetic analyses, where single genes involved in hydrocarbon biosynthesis were disrupted and HC composition altered, further support the role of HCs in desiccation resistance (Chiang et al., 2016; Chung et al., 2014; Qiu et al., 2012). Moreover, plasticity in desiccation resistance in response to environmental stress (i.e. rapid desiccation hardening) has been associated with changes to HC profile and cuticular permeability (Bazinet et al., 2010; Hoffmann, 1990, 1991; Stinziano et al., 2015).

Our previous work established a novel system to test the capacity of HCs to protect against desiccation in *Drosophila melanogaster.* The system utilized engineered flies in which the oenocytes, the integumentary cells responsible for producing HCs, can be genetically ablated (Billeter et al., 2009). Only trace amounts of HCs remain on the cuticle of adult oenocyte-less flies (hereafter referred to as oe− flies; Billeter et al., 2009). Oenocyte ablation was determined to significantly reduce survivorship under desiccative conditions (Krupp et al., 2020). Although increased transpiration was hypothesized to be responsible for the reduced survivorship, this possibility was not directly tested. Here, we examined the primary physiological processes underling desiccation resistance, including (i) water storage (bulk and metabolic water), (ii) water-loss tolerance (i.e. dehydration tolerance) and (iii) water-loss prevention, to determine which, if any, contribute to the reduced survival of oe− flies under desiccative conditions. By employing physiological methods, we demonstrate a direct relationship between oenocytes and HCs and transpiration rate. We propose that the oe− model will provide an important system with which to further explore the crucial role played by the insect cuticle in desiccation resistance.

## MATERIALS AND METHODS

### Fly strains and rearing

Fly strains were reared on food containing agar, glucose, sucrose, yeast, cornmeal, wheat germ, soya flour, molasses, propionic acid and Tegosept on a 12 h:12 h light:dark cycle (LD 12:12) at 25°C and 50% relative humidity. All flies were collected as virgins under CO_2_ anaesthesia and kept in same-sex groups of 50±5 individuals per vial. The Canton-S strain of *D. melanogaster* was used as the wild-type strain. Previously described methods and strains were used to generate flies that lacked adult oenocytes (i.e. oenocyte-less flies; oe−) (Billeter et al., 2009). Adult oe− flies were obtained by crossing ‘+; *PromE(800)-Gal4, tubP-Gal80^ts^*; +’ with ‘+; *UAS-StingerII, UAS-hid*; +’. Genetic background control flies were obtained by crossing ‘+; *PromE(800)-Gal4, tubP-Gal80^ts^*; +’ to ‘+; *UAS-StingerII*; +’. Progeny were raised at 18°C until eclosion to avoid larval lethality. Virgin adults were subjected to a temperature treatment of 25°C for ∼24 h, followed by 30°C for 3 days, and a recovery period of 3–4 days at 25°C. This temperature treatment, referred to as 18°C>30°C>25°C, conditionally restricted the expression of the pro-apoptotic gene *hid* to the adult oenocytes. The completeness of oenocyte ablation was confirmed by the absence of green fluorescent protein (GFP) fluorescence emission (*UAS-stingerII*) in all oe− flies. Gas chromatography (see below) analysis was performed on select oe− flies to ensure the loss of HCs. Wild-type Canton-S and genetic control flies were subjected to the same temperature treatments and rearing conditions as oe− flies. All flies were 10 day old virgins at the time of testing except where noted.

### Desiccation assay

To test desiccation resistance, groups of 50 flies were placed into desiccation chambers and survivorship measured. Each desiccation chamber consisted of two connected narrow-mouthed fly vials (25 mm×95 mm), sealed with laboratory film (Bemis, Parafilm M). Desiccation chambers were placed in an upright position. Grouped flies were housed in the upper vial without access to food or water and separated by a cotton plug from the lower vial containing the desiccant (Drierite, 8 mesh). Flies were loaded into the desiccation chambers using brief cold anaesthesia (chilled for 3–4 min on ice). Desiccation chambers were maintained at 30°C and 50% relative humidity for the duration of the experiment. Survivorship was measured hourly, and the assays ended when 50% of the flies had died (referred to as mortality time; MT50). MT50 determination allowed for accurate water loss measurements to be taken at a distinct time in the survivorship curve – a requirement for quantifying dehydration tolerance and water loss rate (see methods below). Death was determined by lack of movement and an inability to resume an upright position. *n*=11–17 replicate groups for each genotype tested.

### Gravimetric water measurements

Gravimetric water measurements were made using a microbalance (Mettler XS205, Columbus, OH, USA). To determine body water content, individual groups of 50±3 flies were weighed immediately prior to (group wet mass) and after being completely dried (group dry mass; 48 h at 30°C in a desiccation chamber). *n*=5–9 replicate groups for each genotype tested. To determine dehydration tolerance, individual groups of 50±3 flies were weighed immediately prior to desiccation (group wet mass), at the desiccation point at which 50% of the population had reached mortality (group MT50 mass; 30°C in a desiccation chamber), and after being completely dried (group dry mass; 48 h at 30°C in a desiccation chamber). *n*=11–17 replicate groups for each genotype tested.

The following equations were used (final values were divided by the number of flies per group):
(1)

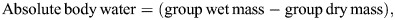

(2)

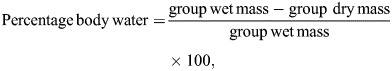

(3)

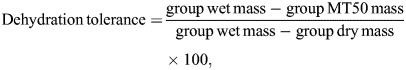
where dehydration tolerance is also referred to as percentage total body water loss at MT50, and
(4)

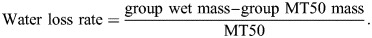


### Glycogen measurement

Metabolic water, or the water released during the metabolism of certain lipids and carbohydrates, is an important reservoir of stored water utilized by animals to prevent desiccation (Djawdan et al., 1998). Glycogen is the primary carbohydrate in *Drosophila* and an important reservoir of metabolic water (Schmidt-Nielsen, 1997). Glycogen measurements were adapted from Tennessen et al. (2014). Briefly, groups of 5 flies were homogenized in PBS (100 µl) in 1.5 ml microcentrifuge tubes using pellet pestles. To control for sample variability, 30 µl of each sample was reserved for protein quantification [Bradford reagent (Sigma; B6916) and Protein Standard (BSA) Solution (Sigma; P0834), used according to the manufacturer's instructions]. Remaining sample volumes were heat treated (10 min at 70°C) and diluted (1:6) with either PBS alone to measure free glucose or PBS plus amyloglucosidase (AS; Sigma; A1602) to measure total glucose, including both free glucose and glucose metabolized from glycogen. The difference between total glucose and free glucose represented the amount of glucose stored as glycogen. Glucose levels were measured using the HK glucose assay reagent (Sigma; GAHK20). Absorbance readings (340 nm) were made using a microplate reader (BioTek). Glucose amounts were determined by comparison to a standard curve of known glucose concentrations and normalized against protein amount (see above). Assays with glycogen standards were performed in tandem with experimental samples to control for the completeness of the AS digestion (Sigma; G0885). *n*=4 replicate groups for each genotype tested.

### Water loss rate measurements

Water loss rate was measured empirically using flow-through respirometry. The recording period lasted 1 h from the point that the insect chamber was connected to the gas analyser, during which CO_2_ and H_2_O measurements were collected every second. Once connected, the ambient chamber air was immediately flushed with 150 ml min^−1^ dry CO_2_-free air for 2 min to remove any residual environmental water vapour. The chamber-housed flies were allowed to acclimate for a total of 30 min. The 30 min mark became the set start time for a 5 min recording interval used for analysis. The set start time of this 5 min interval was held constant across all replicate runs and specifically chosen to ensure a stable recording period while all flies of the different genotypes were at their healthiest following the necessary flushing/acclimation period. Average H_2_O and CO_2_ levels (expressed as ppt fly^−1^ min^−1^ and ppm fly^−1^ min^−1^, respectively) were determined from the 5 min recording. Baseline measurements were acquired from an empty chamber without flies (but otherwise prepared as described directly above) before and after each experimental run. The average of the two baseline measurements was subtracted from the experimental measurement to remove background noise and correct for instrument drift. *n*=4 replicate groups for each genotype tested.

Results obtained by flow-through respirometry were supported by a complementary gravimetric method. Gravimetric measurements were used to calculate water loss rate according to the equation *R*=*S*/*T*, where *R* represents the water loss rate (in mg fly^−1^ s^−1^), *S* is the absolute water lost at death (in mg fly^−1^), and *T* is the time at MT50 (in s). *n*=11-17 replicate groups for each genotype tested.

### Cohabitation and mating conditions

HCs are transferred between flies during copulation. Virgin oe− flies were housed with the opposite sex virgin wild-type flies in groups of 100 flies at a 1:1 sex ratio and maintained at 25°C and 50% relative humidity. Flies were provided with unrestricted opportunities to interact and mate. Initial mating events were directly observed when possible. Wild-type and oe− females always mated within the first 30 min of being introduced to wild-type males. Any subsequent re-matings beyond the initial 30 min observation period were not monitored. oe− males are not attractive to females and exhibit a prolonged mating duration (Billeter et al., 2009). Mating events of oe− males were not confirmed. Two cohabitation conditions were used: (i) 24 h housed together continuously and (ii) 24 h housed together followed by a 24 h recovery period in which flies were housed separately (referred to as 24 h>>24 h). At the end of the designated cohabitation conditions, mated oe− flies were separated using CO_2_ anaesthesia and the water loss rate of oe− flies was immediately measured by flow-through respirometry as described above. Virgin control flies were subjected to identical treatment. *n*=4–7 replicate groups for each condition tested.

### Cuticular hydrocarbon extraction

Whole fly cuticular HC extract was derived separately from 7–8 day old Canton-S virgin male and female flies; 400 flies were cold-anesthetized and placed in a 5 ml vial containing 1.6 ml of hexane. To ensure effective extraction, vials were gently vortexed for 2 min. HC extracts were pooled from replicate batches to create a ‘master HC extract’, which was utilized for all subsequent coating experiments. Roughly a 5-fly equivalent of extract was required to coat a single oe− fly with approximately wild-type levels of HCs.

### Coating protocol

An amount of ‘master HC extract’, empirically determined to coat a group of 50 flies with near-wild-type levels, was dispensed into individual 1.5 ml vials and the volume increased to 400 μl with additional hexane. Approximately a 250-fly equivalent of HC extract was required to coat a group of 50 flies. The hexane solvent was evaporated under nitrogen gas, leaving behind a vial with an inner surface coated with the test HC. Groups of 50 oe− flies were placed into pre-treated vials and gently vortexed 3 times for 20 s with pauses of 20 s. Mock control flies were treated identically except vials were pre-treated with hexane only. Following the coating treatment, coated and mock control flies were immediately used for water loss rate experiments. *n*=3 replicate trials for each condition tested. Two to four flies from each treatment vial were withheld and immediately tested to confirm the amount transferred using the cuticular HC analysis described below. The cuticular HC profiles of coated oe− flies were compared with those of Canton-S flies.

### Cuticular hydrocarbon analysis

Flies were cold anesthetized and individually placed into glass micro-vials containing 50 ml of hexane containing 10 ng ml^−1^ of octadecane (C18) and 10 ng ml^−1^ of hexacosane (C26) as injection standards. To achieve efficient extraction, micro-vials were gently agitated for 2 min. HC extracts were analysed using an Agilent 7890A gas chromatograph system with a flame ionization detector (GC/FID) and PTV injector (cool-on-column mode) and outfitted with a DB-1 20 m×0.18 mm Agilent 121-1022 fused silica capillary column (Agilent Technologies, Inc., Santa Clara, CA, USA) as previously described (Krupp et al., 2013). Sample volumes of 1 μl were injected into the column. Helium was the carrier gas and was applied at a constant flow rate of 1 ml min^−1^. Analysis of the extract was carried out with a column temperature profile that began at 50°C (held for 1 min) and was ramped at 36.6°C min^−1^ to 150°C and then at 5°C min^−1^ to 280°C, where it was held for 10 min. The injector and FID temperatures were programmed to 280°C and 300°C, respectively. Agilent OpenLAB CDS (EZChrom Edition) software was used to calculate the retention time and total area of each peak. The mass of each HC extracted from an individual fly was calculated by dividing the area of each peak by that of the corresponding peak of the C26 internal standard and multiplying by the initial extract volume.

### Statistical analysis

All statistical analyses were performed using PRISM 9.5.1 (GraphPad, San Diego, CA, USA). Two-way ANOVA with *post hoc* Tukey's multiple comparison test was used to determine significance (α=0.5) unless otherwise indicated. Principal component (PC) analysis was performed on rescued oe− flies coated with wild-type HC extract using the water loss rate data (see below and Table S1). Individual HC compounds were summated according to chemical class [i.e. *n*-alkane, 2-methyl alkane, (*Z*)-alkene and (*Z,Z*)-alkadiene]. Variables included the average amounts of the four chemical classes, the total amount of all HCs and water-loss rate. Components were selected that together accounted for >95% of the total variance. In all instances, this included only PC1 and PC2.

## RESULTS

### Oenocytes are required for desiccation survival

**Fig. 1.**
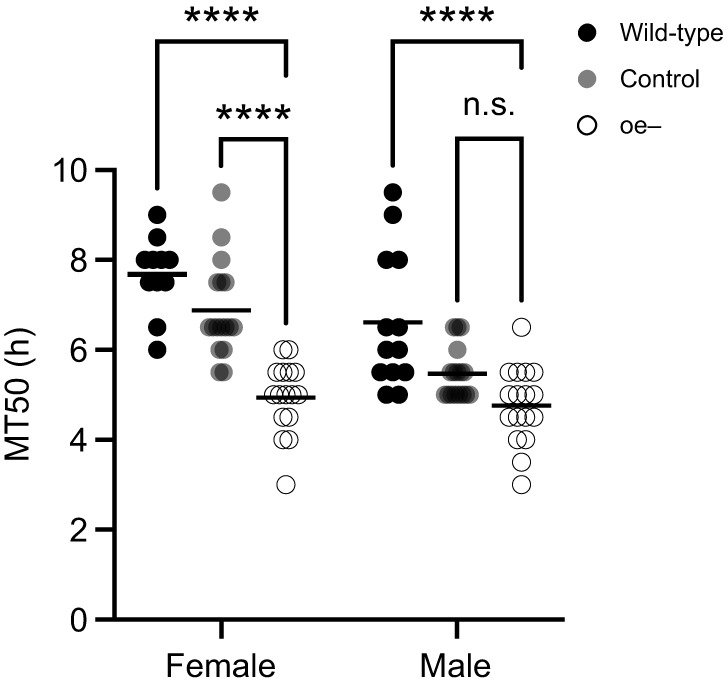
**Oenocytes are required for desiccation survival.** Mean time for 50% group mortality (MT50) for females and males under desiccative conditions at 30°C. Each data point represents an independent biological replicate, each consisting of a group of 50 flies housed in a separate desiccation chamber for females (wild-type, *n*=11: control, *n*=17; oe−, *n*=16) and males (wild-type, *n*=13; control, *n*=17; oe−, *n*=17). Two-way ANOVA with Tukey's test (****P<0.0001; n.s., not significantly different). Horizontal black bar represents the sample mean for the indicated measurement.

The survivorship of oe− flies was previously observed to be significantly compromised under desiccative conditions at 25°C, exhibiting a median survival time less than half that of control groups (Krupp et al., 2020). Here, we confirmed our previous results using a different measure of survival, MT50 or the time at which the group population reached 50% mortality. We found a statistically significant difference in average MT50 by both sex (*F*_1,85_=18.54, *P<*0.0001; Fig. 1) and genotype (*F*_2,85_=40.67, *P<*0.0001; Fig. 1). When compared by sex, both wild-type and control females survived significantly longer than their male counterparts (*P*=0.0549 and *P*=0.0007 respectively; Fig. 1). Interestingly, a similar sexual dimorphism between oe− female and male flies was not observed (*P*=0.9955; Fig. 1). When compared by genotype, the average MT50 for oe− females was significantly reduced compared with that of wild-type and control females (*P*<0.0001; Fig. 1), while that for oe− males was significantly reduced compared with that of wild-type males (*P*<0.0001; Fig. 1) but not compared with the control (*P*=0.1125; Fig. 1).

### Oenocyte ablation produces a sex-specific difference in water storage

**Fig. 2.**
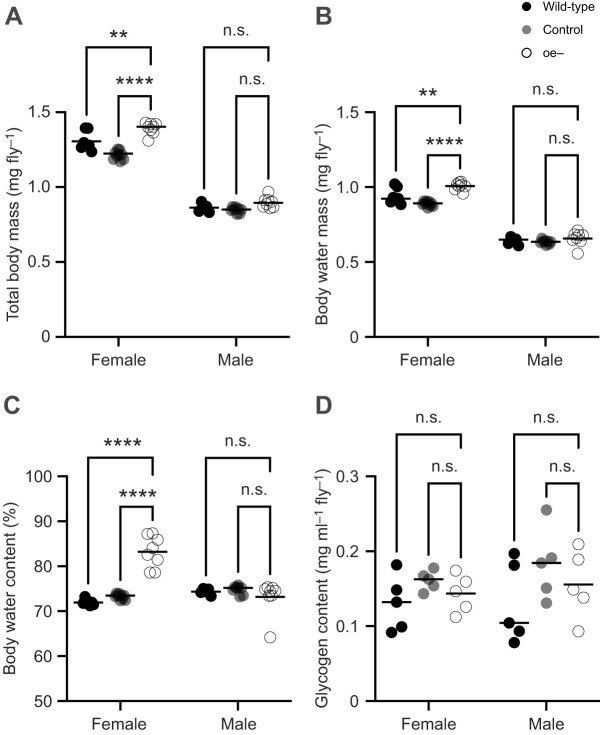
**Oenocyte ablation produces a sex-specific difference in water storage.** (A) Total body mass determined as a metric for body size. The mean total body mass of oe− females is significantly higher than that of the control and wild-type females. (B) Absolute body water determined gravimetrically. The mean absolute body water of oe− females is significantly higher than that of the control and wild-type females. (C) Percentage body water determined gravimetrically and calculated relative to the total body mass. The mean body water percentage of oe− females is significantly higher than that of the control and wild-type females. Data acquired from the same groups is presented in A, B and C. Each data point represents an independent biological replicate for females (wild-type, *n*=7; control, *n*=9; oe−, *n*=8) and males (wild-type, *n*=5; control, *n*=8; oe−, *n*=8). (D) Glycogen content representing the amount of metabolic water per fly. Each data point represents an independent biological replicate for females and males (wild-type, *n*=5; control, *n*=5; oe−, *n*=5). Two-way ANOVA with Tukey's test (****P<0.0001; **P<0.01; n.s., not significantly different). Horizontal black bar represents the sample mean for the indicated measurement.

The reduced desiccation survival of oe− flies could be the result of an inability to acquire or store sufficient water to offset desiccation. To investigate this possibility, the amounts of both total body water and metabolic water stores were quantified and compared across genotypes. Gravimetric measurements of mass were used to determine body water content. Female flies, in general, exhibited significantly greater total body mass (*F*_1,39_=1441, *P<*0.0001; Fig. 2A) and absolute body water mass (*F*_1,39_=920.9, *P<*0.0001; Fig. 2B) than males across all genotypes. This is attributed to a sexual dimorphism in body size, with females being the larger of the sexes. Accordingly, when body water mass was expressed as a percentage of total body mass (i.e. percentage body water), both wild-type and control flies no longer exhibited a sex difference (Fig. 2C).

Yet, when comparing by genotype, oe− females displayed a significantly higher total body mass than sex-matched wild-type and control flies (*P*<0.0001 and *P*=0.0011, respectively; Fig. 2A). oe− females also displayed a significantly higher absolute body water mass than wild-type and control females (*P*=0.0042 and *P*<0.0001, respectively; Fig. 2B). Correspondingly, oe− females also showed a significantly higher body water percentage compared with both wild-type and control females (*P*<0.0001; Fig. 2C). oe− males, in contrast, were not different from wild-type (*P*=0.9565; Fig. 2C) or control males (*P=*0.7192; Fig. 2C) in percentage body water, or in any other measure (Fig. 2A,B).

Glycogen is the primary carbohydrate in *Drosophila* and an important reservoir of metabolic water (Schmidt-Nielsen, 1997). Measurements of glycogen were made to determine the potential availability of metabolic water in oe− and control flies. Glycogen content was not significantly different by sex (*F*_1,24_=0.5999, *P*=0.4462; Fig. 2D) or by genotype (*F*_2,24_=2.685, *P*=0.0887; Fig. 2D). Together, these results indicate that deficient water storage is unlikely to account for the reduce survivorship of oe− flies under desiccative conditions.

### Oenocytes do not affect dehydration tolerance

**Fig. 3.**
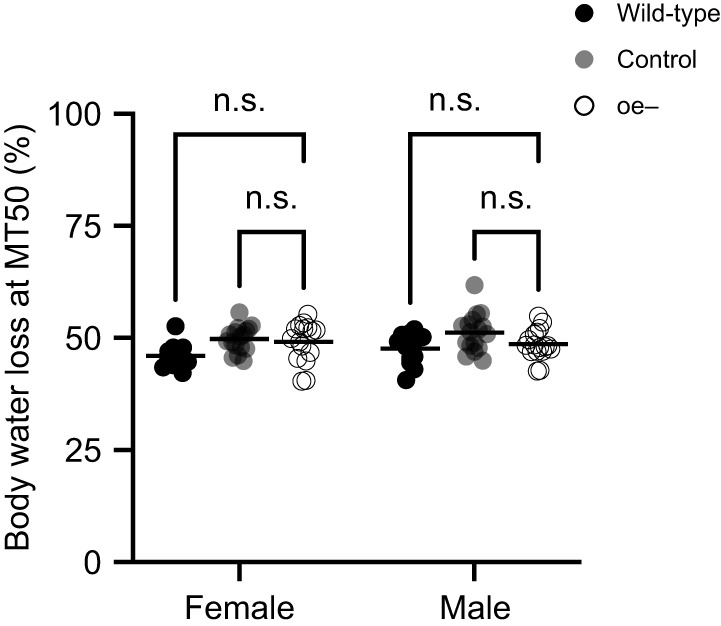
**Oenocytes do not affect dehydration tolerance.** Dehydration tolerance represented as the mean percentage of total body water loss at MT50. Each data point represents an independent biological replicate, each consisting of a group of 50 flies for females (wild-type, *n*=11; control, *n*=17; oe−, *n*=16) and males (wild-type, *n*=13; control, *n*=17; oe−, *n*=17). Two-way ANOVA with Tukey's test (n.s., not significantly different). Horizontal black bar represents the sample mean for the indicated measurement.

Desiccation tolerance or the degree to which an organism can withstand dehydration, impacts survival under desiccative conditions. Dehydration tolerance was not significantly different by sex (*F*_1,85_=1.231, *P=*0.2703; Fig. 3) or genotype (*F*_2,85_=7.203, *P=*0.0013; Fig. 3). Thus, a decrease in dehydration tolerance is unlikely to explain the reduced survival of oe− flies.

### Oenocytes are required to prevent evaporative water loss

**Fig. 4.**
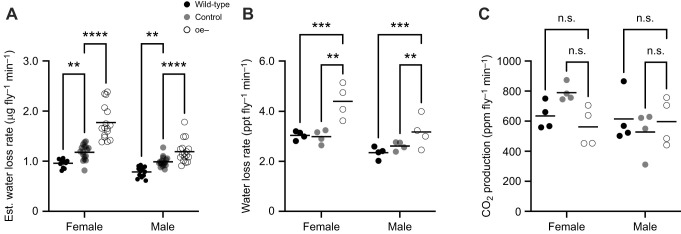
**Oenocytes are required to prevent evaporative water loss.** (A) Mean water loss rate for females and males estimated using measurements of body water mass and time to MT50. Each data point represents an independent biological replicate consisting of a group of 50 flies for females (wild-type, *n*=11; control, *n*=17; oe−, *n*=16) and males (wild-type, *n*=13; control, *n*=17; oe−, *n*=17). (B,C) Mean rates of water loss (B) and CO_2_ production (C) for females and males as determined by flow-through respirometry. Each data point represents an independent biological replicate consisting of a group of 50 flies for both females and males (wild-type, *n*=4; control, *n*=4; oe−, *n*=4). ppt, parts per thousand; ppm, parts per million. Two-way ANOVA with Tukey's test (****P<0.0001; ***P<0.001; **P<0.01; n.s., not significantly different). Horizontal black bar represents the sample mean for the indicated measurement.

Next, we investigated whether the reduced survival of oe− flies under desiccative conditions was linked to an elevated rate of evaporative water loss. To do so, we first estimated the rate of transpiration using measurements of body water mass and time to MT50 (see Materials and Methods). Our analysis revealed that the estimated rate of water loss under desiccative conditions was significantly different between the sexes (*F*_1,87_=50.87, *P<*0.0001; Fig. 4A) and across genotypes (*F*_2,87_=56.59, *P<*0.0001; Fig. 4A). The estimated water loss rate for oe− females was significantly higher (∼1.7-fold) than that for wild-type and control flies (*P*<0.0001; Fig. 4A). Similarly, the estimated rate for oe− males was significantly higher (∼1.4-fold) than that for both wild-type and control flies (*P*<0.0001; Fig. 4A).

The calculated rate estimates above were confirmed by the direct measurement of water loss using flow-through respirometry. The rate of water loss under desiccative conditions was found to be significantly different between the sexes (*F*_1,20_=17.86, *P<*0.0004; Fig. 4B) and across genotypes (*F*_2,20_=14.77, *P<*0.0001; Fig. 4B). Comparable to estimates above, we found that the water loss rate of females was significantly higher compared with that of males independent of genotype (*F*_1,87_=50.87, *P<*0.0001; Fig. 4B). Moreover, the water loss rate of oe− females was significantly higher (∼1.5-fold) than that of both wild-type and control females (*P*=0.0010 and *P*=0.0029, respectively; Fig. 4B). Similarly, the water loss rate of oe− males was significantly higher (∼1.3-fold) compared with that of the wild-type and control flies (*P*=0.0010 and *P*=0.0029, respectively; Fig. 4B).

Water loss is also associated with respiration. The respiratory rate of oe− flies was determined directly by measuring CO_2_ levels using flow-through respirometry. Our results revealed no significant differences in the rate of CO_2_ production either between the sexes (*F*_1,18_=2.340, *P=*0.1434; Fig. 4C) or across genotypes (*F*_2,18_=0.7286, *P=*0.4963; Fig. 4C). Together, these results demonstrate that the oenocytes are critical for restricting the rate of water loss due to transpiration.

### Cuticular hydrocarbons are required to prevent evaporative water loss

**Fig. 5.**
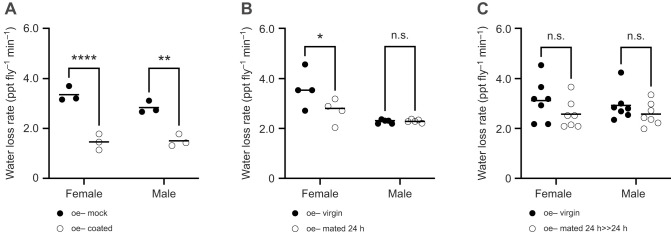
**Cuticular hydrocarbons (HCs) are required to prevent evaporative water loss.** (A) Mean water loss rate for oe− flies coated with sex-specific wild-type HC extract as determined by flow-through respirometry. The amount of cuticular HCs coating the oe− flies was equivalent to that typically found on an average wild-type fly. Mock control flies were treated identically to experimental flies but did not receive HC extract. Each data point represents an independent replicate for both females and males (oe− mock, *n*=3; oe− coated, *n*=3). (B,C) Mean water loss rate for oe− flies following cohabitation with wild-type flies of the opposite sex (sex ratio of 1:1) for the indicated periods of cohabitation (24 h or 24 h>>24 h) as determined by flow-through respirometry. The decreased water loss rate in females after cohabitation (24 h; B) is due to the direct transfer of HCs from wild-type males. Each data point represents an independent replicate for females (oe− virgin, *n*=4; oe− mated, *n*=4) and males (oe− virgin, *n*=5; oe− mated, *n*=5). No change in water loss rate was observed after transferred HCs were allowed to dissipate for 24 h following cohabitation (24 h>>24 h; C). Each data point represents an independent biological replicate (oe− virgin, *n*=7; oe− mated, *n*=7) for both females and males. Two-way ANOVA with Tukey's HSD test (****P<0.0001; **P<0.01; *P<0.05; n.s., not significantly different). Horizontal black bar represents the sample mean for the indicated measurement.

We next asked whether restoring HCs to oe− flies would also rescue the rate of evaporative water loss. To do so, whole HC extracts derived separately from virgin wild-type females and males were topically applied to the cuticle of sex-matched oe− flies (Table S1). oe− female and male flies coated with wild-type HC extracts had significantly reduced rates of water loss compared with uncoated oe− mock control flies. oe− females coated with female HC extract had a 3-fold decrease in water loss rate compared with the mock female control flies (*F*_1,8_=101.4, *P*<0.0001; Fig. 5A), and oe− males coated with male HC extract had a 2-fold decrease in water loss rate compared with the male control flies (*P*=0.0017; Fig. 5A). The rate of water loss was not significantly different by sex (*F*_1,8_=2.122, *P=*0.1833; Fig. 5A). Principal component analysis revealed a strong negative correlation between all classes of cuticular HCs and water loss rate within PC1, most likely reflecting the presence (or amount) of HCs (Fig. S1A–D). To a lesser degree, a positive correlation was found within PC2, possibly reflecting differences in the chemical properties of the individual HC classes to protect against water loss (Fig. S1A–D). These correlations were generally consistent between females and males. Together, these results demonstrate that HCs are both necessary and sufficient to protect against water loss through the cuticle.

Next, we sought to test whether the transfer of HCs previously shown to occur between flies during copulation is sufficient to affect evaporative water loss. oe− females were permitted to cohabitate and freely associate with wild-type males for 24 h during which time the flies copulate (likely multiple times), thus facilitating the transfer of wild-type HCs to the oe− female. The water loss rate was significantly different by sex (*F*_1,14_=18.58, *P=*0.0007; Fig. 5B) or mated condition (*F*_1,14_=4.647, *P*=0.0490; Fig. 5B). Mated oe− females showed a significant decrease in water loss rate compared with oe− virgin females (*P*=0.0442; Fig. 5B). In contrast, oe− males showed no change in water loss rate after cohabitation with wild-type females compared with oe− virgin males (*P*=0.9997; Fig. 5B). This is likely because oe− males lacking HCs, many of which are potent sex pheromones, are generally unattractive to females. Therefore, oe− males may not have successfully copulated and thus missed the opportunity to acquire HCs. When a 24 h recovery period immediately following cohabitation but prior to testing was provided to allow transferred HCs to dissipate, water loss rate was no longer significantly different by sex (*F*_1,24_=3.434, *P*=0.0762; Fig. 5C) or by mated condition (*F*_1,24_=0.1585, *P*=0.6941; Fig. 5C). Together, these results reveal that oe− females receive a short-term (<24 h) protective advantage in desiccation resistance via the transfer of HCs to the cuticle during copulation with wild-type flies.

## DISCUSSION

Here we sought to identify the physiological mechanism(s) responsible for the decreased survivorship of oe− flies under desiccative conditions. To do so, we measured water storage, dehydration tolerance, and respiratory and transpiratory water loss of both female and male flies. Perhaps in the clearest demonstration yet, our results show that insect cuticular HCs are critically important for protecting against evaporative water loss and dehydration.

### Oenocytes and body water stores

Body size affects both water storage and transpiration. While a larger body size is potentially beneficial, allowing for greater water storage (either as bulk or metabolic water reserves) and reduced transpiration rate, it also exerts greater physiological demands that may negatively impact desiccation resistance (i.e. increased metabolic and respiratory rates) (Lehmann, 2002; Lehmann and Schützner, 2010). Sexual dimorphisms in body size and mass are well documented in *Drosophila*, where females are typically larger and weigh more than males (Gibbs et al., 2003). The innate size difference is thought to contribute to the greater desiccation resistance consistently observed in females across numerous studies (Chown et al., 2011; Gibbs and Matzkin, 2001). Comparative analysis of over 80 *Drosophila* species largely supports the link between sex-based differences in body size and desiccation resistance (Kellermann et al., 2022). In most cases, larger body size correlates with enhanced desiccation resistance; however, exceptions exist. For example, *Drosophila mojavensis* males, which inhabit the arid Mojave Desert, exhibit greater desiccation resistance despite a smaller size (Matzkin et al., 2007). Similarly, *Drosophila hydei* males, which inhabit cold and dry high-altitude regions, also display greater desiccation resistance despite being smaller than females (Kalra and Parkash, 2014). However, it is important to note that a proportional sex difference in size within the larger *D. hydei* species (relative to the male–female difference in *D. melanogaster*) would be expected to contribute less to the sexual dimorphism in desiccation tolerance. Nonetheless, while body size clearly plays a significant role, these exceptions highlight the fact that other physiological and behavioural factors contribute to differences in desiccation resistance.

Consistent with previous studies, we demonstrate that oe− female flies have a greater body mass and body water mass than oe− male flies (Fig. 2A,B). This aligns with a greater body water mass observed generally in females across different genotypes and species. Surprisingly, oe− females also showed an elevated body water percentage above that of control females; approximately 10% greater than background control and 12% greater than wild-type (Fig. 2B). oe− males, in contrast, did not exhibit any measurable difference in body mass or water content compared with the control groups (Fig. 2A–C). Nonetheless, both oe− females and males displayed reduced survival under desiccative conditions (Fig. 1). These findings are supported by a recent study conducted by Ferveur et al. (2018) which utilized transgenic flies to knockdown the expression of the *desat1* gene, a gene that encodes a desaturase enzyme integrally involved in the biosynthesis of unsaturated HCs. The oenocyte-targeted knockdown of *desat1* expression resulted in elevated water content yet reduced desiccation resistance in females (Ferveur et al., 2018). Together with our findings, these results suggest that the increased water storage observed in oe− females, though warranting further study, is unable to compensate for an elevated state of dehydration, and that the relationship between oenocyte function and HC biosynthesis (and perhaps lipid metabolism, in general) supports other physiological mechanisms important for desiccation resistance. Indeed, in addition to HC biosynthesis, oenocytes have been shown to play an important role in lipid mobilization and metabolic homeostasis (Chatterjee et al., 2014). Perhaps this function affects metabolic water storage or some other yet to be identified mechanism involved in water balance.

### Oenocytes and dehydration tolerance

Under desiccative conditions, *Drosophila*, like all other insects, can endure a certain level of water loss before dying, a phenomenon referred to as dehydration tolerance. Comparative studies have shown variable differences in dehydration tolerance within species and across ecological habitats (Gibbs and Matzkin, 2001; Telonis-Scott et al., 2006). While the mechanisms underlying dehydration tolerance are not fully understood, it is known to involve multiple physiological and biochemical processes, including water retention, cellular protection, osmoregulation and metabolic modulation (Gibbs, 2002a,b; Thorat and Nath, 2018). For example, the coordinated regulation of extracellular haemolymph volume, a crucial factor in maintaining osmoregulation, can provide the fly with extra survival time under conditions of water scarcity (Beyenbach, 2016; Folk and Bradley, 2003). Relatedly, glycogen, an important reservoir of metabolic water, can be metabolized to provide body water to cope with dehydration. Notably, flies artificially selected for desiccation resistance consistently exhibited elevated glycogen stores. (Chippindale et al., 1998; Djawdan et al., 1998; Gibbs and Matzkin, 2001).

Measured as the percentage body water lost, measured at the point of death, the dehydration tolerance of oe− flies was not different from that of controls. All flies independent of genotype and sex tolerated the loss of approximately 50% of total body water. Similarly, the glycogen content of oe− flies was not different from that of controls. Together, this strongly suggests that oenocytes, though important for lipid metabolism (Huang et al., 2022), do not contribute substantially to the processes that mediate desiccation tolerance, at least under the extreme desiccative conditions tested here.

### Transpiratory and respiratory water loss

Transpiration and respiration represent the primary routes of water loss in *Drosophila*, respectively estimated to account for ∼70% and ∼25% of water lost to the environment under desiccative conditions (Wang et al., 2021). While transpiration is a passive process involving the evaporative loss of water across the cuticle, respiration involves the regulated exchange of gases and water between the external environment and the interior tracheal system.

Transpiration has been clearly shown to be affected by HCs (Gibbs and Rajpurohit, 2010; Rajpurohit et al., 2008; Wang et al., 2021). Many studies have documented the link between the HCs and cuticular permeability (Chiang et al., 2016; Ferveur et al., 2018; Gibbs, 2002a,b; Horváth et al., 2023; Qiu et al., 2012; Wang et al., 2022). HC amount and composition, chain length and saturation level are all factors that have been studied in relation to cuticular permeability (Gibbs, 2002a; Gibbs and Pomonis, 1995). Cuticular permeability can also be altered through structural modifications to the cuticle, including melanization (Kalra et al., 2014); however, the mechanisms are still unclear. Melanin granules are hydrophobic and may reduce the cuticular permeability through interactions with cuticular proteins (Kalra et al., 2014). Differences in cuticular melanization levels correlate with variation in both cuticular permeability and desiccation resistance (Parkash et al., 2009; Rajpurohit et al., 2008; Ramniwas et al., 2013). The degree of melanization of oe− flies has not been determined.

Respiration has also been a source of vigorous research in *Drosophila*. Regulated by the active opening and closing of the cuticular spiracles, flies utilize various respiratory patterns that reflect the metabolic demands of differing physiological and behavioural states, and environmental conditions (Lehmann and Schützner, 2010; Quinlan and Gibbs, 2006; Williams et al., 1997). The discontinuous gas exchange (DGE) cycle, in which spiracles periodically open and close to allow the intermittent exchange of gases, aids in conserving water, particularly in flies at rest (Gibbs et al., 2003). In contrast, the continuous gas exchange (CGE) cycle, in which the spiracles are held open, ensures a more consistent exchange of gases while flies are engaged in energy-intensive activities (Lehmann, 2001; Lehmann and Schützner, 2010). Perhaps not surprisingly, flies selected for increased desiccation resistance exhibit longer and more frequent cycles of DGC, thus limiting water loss (Williams and Bradley, 1998; Williams et al., 1997).

We used two methods to quantify the transpiratory and respiratory rates of oe− flies: (i) indirect gravimetric measurements and (ii) direct flow-through respirometry analysis. As anticipated, both oe− female and male flies exhibited elevated transpiration rates relative to control groups (Fig. 4A,B). Furthermore, the application of a wild-type HC mixture successfully reduced the water loss rate in oe− females and males compared with the control mock flies (Fig. 5A) and fully restored the water loss rate to control group levels (Fig. 4B). Importantly, respiratory rates (CO_2_ production) were unchanged between oe− and control flies (Fig. 4C), thus indicating that anaerobic respiration does not contribute to the difference in water loss rates between them. It is worth noting, however, that our respirometry measurements were not sensitive enough to determine whether oe− flies exhibited changes to respiratory (DGC versus CGC) or excretory patterns. Together, our findings indicate that increased transpiration resulting from the absence of oenocytes is the primary (and perhaps only) factor negatively influencing the desiccation resistance in oe− flies and represent direct evidence of the critically important role HCs play in reducing evaporative water loss rate.

### Post-mating increase in desiccation resistance

Physical interactions have been shown to facilitate the transfer of HCs between flies when co-housed in a group setting (Krupp et al., 2020). Here, we found that physical interactions occurring during courtship and copulation facilitated the unidirectional transfer of HCs from wild-type males to oe− females, resulting in a significant improvement in desiccation resistance (reduced transpiration rate) in oe− flies (Fig. 5B). However, likely as a result of the limited amount and uneven distribution of transferred HCs, the reduction in water loss rate remained less than that observed in the HC rescue experiments (Fig. 5A). Nonetheless, this result indicates that the transfer of HCs may reduce the risk of dehydration under certain ecological conditions.

Mated oe− female flies have also been shown to exhibit a continued (long-lasting) improvement in desiccation survival (compared with virgin flies) even after the dissipation of transferred HCs (Krupp et al., 2020). As stated above, we found that the reduced transpiration rate of mated oe− females was dependent on the presence of transferred HCs. When transferred HCs were allowed to dissipate for 24 h, the transpiration rate returned to near-virgin oe− female levels (Fig. 5C). Together, these results suggest that while transferred HCs confer a short-term benefit by limiting water loss by transpiration, a yet unidentified post-mating process produces a long-lasting effect on female desiccation survival.

Mated female flies are known to undergo changes in physiology and behaviour that promote the production of offspring, a phenomenon referred to as the post-mating response (PMR). PMR is dependent upon the transfer of male seminal fluid to the female. *Drosophila* seminal fluid contains a number of signalling peptides able to induce changes in many female reproductive processes, including mating receptivity (Liu and Kubli, 2003), egg laying (Kubli, 2003), feeding (Carvalho et al., 2006), immunity (Peng et al., 2005), activity and sleep patterns (Isaac et al., 2010), and importantly, water balance and water retention (Cognigni et al., 2011). Recent genomic studies in *Drosophila* have revealed insights into the genetics of PMR. In the days following copulation, numerous genes exhibit a change in expression pattern (either induced or repressed). A peak is observed on approximately the third day post-mating, and the changes persist for about 7 days. Several PMR genes have been identified that play roles in nutrient homeostasis and metabolism, with their expression patterns showing spatial and temporal changes (Delbare et al., 2023; Newell et al., 2020). It is likely that these PMR-related changes in gene expression drive the persistent effects on desiccation survival of mated oe− females.

To conclude, our findings demonstrate that oenocyte-derived cuticular HCs are indispensable for the prevention of evaporative water loss, and thus critically important to the desiccation resistance of *D. melanogaster*. While autogenously produced compounds represent the bulk of cuticular HCs, heterogeneously acquired compounds from other individuals during social interactions, such as during copulation, may play a supporting role in preventing transpiration. The mix of HCs on the cuticular surface is composed of many different compounds each with unique chemical properties. How individual compounds contribute to the protective properties of the mix is not known. Utilizing the oe− fly model to further examine the capacity of individual HC compounds to prevent evaporative water loss will enhance our understanding of physiological and behavioural mechanisms involved in insect desiccation resistance.

## Supplementary Material

10.1242/jexbio.247752_sup1 Supplementary information
