# Supplementary material for: Cuticular hydrocarbons promote desiccation resistance by preventing transpiration in Drosophila melanogaster
Source: J Exp Biol. 2024 Nov 28;227(23):jeb247752. doi: 10.1242/jeb.247752 (PMC11634026; doi:10.1242/jeb.247752)
Supplement: Supplementary information [file jexbio-227-247752-s1.pdf]

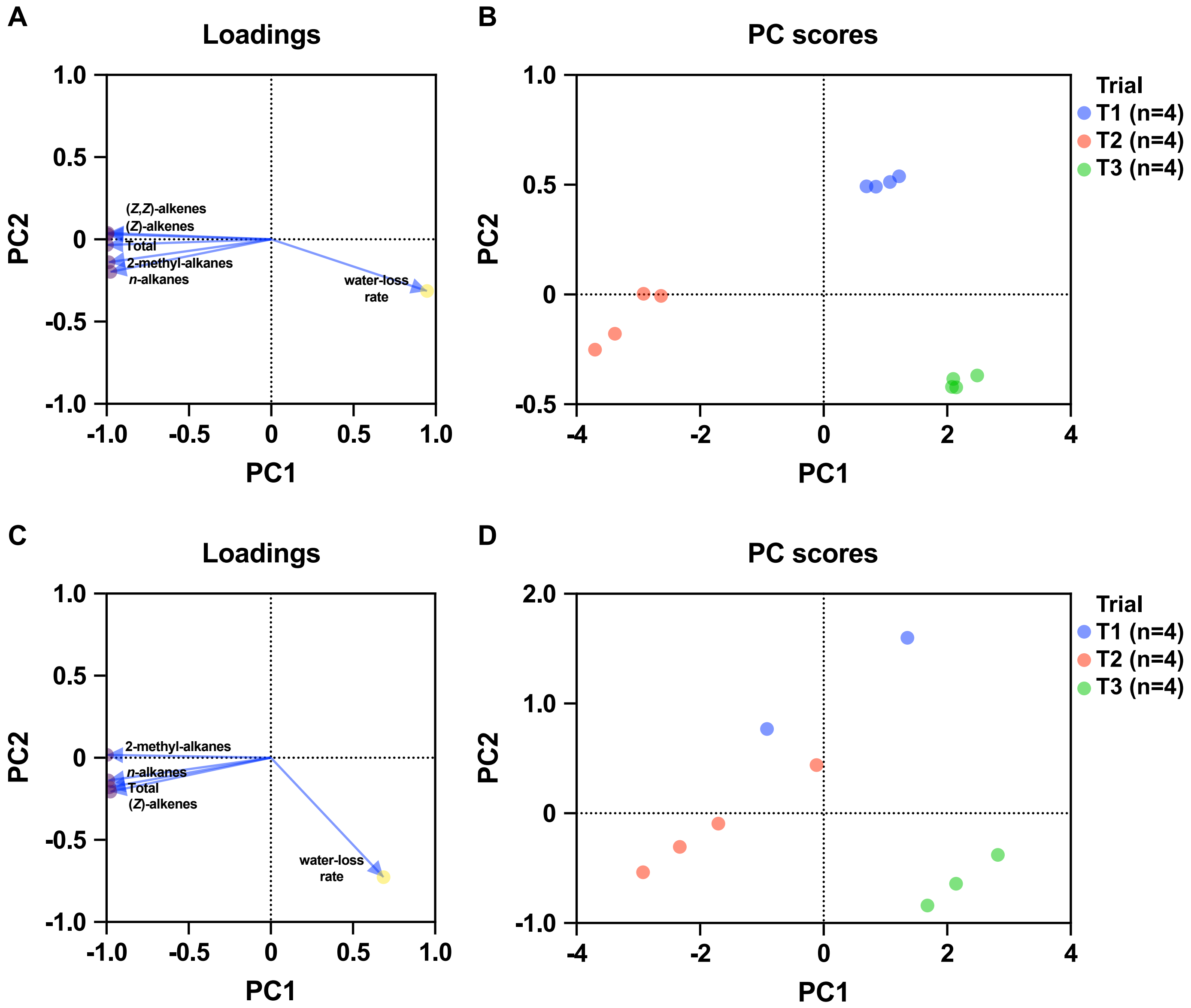

Fig. S1. Principal component analysis of rescued oe- flies. (A-D) Loading and PC score plots for hydrocarbon rescued oe- females (top row) and oe- males (bottom row). **(A,C)** Loading plots show a strong negative correlation between all classes of cuticular hydrocarbons and water loss-rate within PC1, and to a lesser degree, a positive correlation within PC2. Correlations are consistent across both females and males. **(B,D)** HC-coated flies represented in the different rescue trials were clearly distinguishable by PC scores. Variables included the quantified amounts of *n*-alkanes, 2-methyl-alkanes, (*Z*)-alkenes, (*Z,Z*)-alkadienes (females only), total HCs, and water-loss rates of coated rescued oe- flies.

Journal of Experimental Biology • Supplementary information

Table S1. Cuticular hydrocarbon compounds restore desiccation resistance to oenocyte-less flies. Quantification of cuticular HCs transferred to oeflies *via* coating process (see Fig. 5). Transferred HCs were extracted and verified immediately following the coatin g process. Sex-specific wild-type cuticular HC extract was used to coat oe- flies. The amounts of cuticular HCs are represented as the sum values of the four chemical classes of cuticular HCs. All values are in ng. Values in parentheses represent ±SEM. na, not applicable

		Cuticular Hydrocarbon Class					
		**∑*n*-alkanes**	**∑2Me-alkanes**	**∑(*Z*)-alkenes**	**∑(*Z,Z*)-alkadienes**	**total**	***n***
Canton-S - female (wild type)		456.8 (50.0)	236.4 (9.7)	722.9 (62.5)	895.8 (159.7)	2316.4 (257.6)	15
oenocyte-less - female (HC coated)	Trial 1	273.1 (12.2)	206.5 (5.8)	749.0 (45.2)	1508.2 (71.2)	2736.8 (132.8)	4
oenocyte-less - female (HC coated)	Trial 2	1199.5 (153.4)	711.0 (387.0)	1447.9 (155.3)	3129.3 (146.5)	6487.6 (820.9)	4
oenocyte-less - female (HC coated)	Trial 3	255.1 (6.4)	156.6 (9.2)	495.0 (11.1)	882.4 (108.1)	1789.2 (130.6)	4
		**∑*n*-alkanes**	**∑2Me-alkanes**	**∑(*Z*)-alkenes**	**∑(*Z,Z*)-alkadienes**	**total**	***n***
Canton-S - male (wild type)		397.7 (25.7)	182.1 (11.4)	1507.0 (157.9)	na	2086.7 (189.8)	14
oenocyte-less - male (HC coated)	Trial 1	296.5 (55.7)	113.0 (20.9)	1619.6 (297.5)	na	2029.1 (374.1)	2
oenocyte-less - male (HC coated)	Trial 2	448.0 (45.1)	168.5 (18.1)	2425.5 (199.1)	na	3042.0 (262.0)	4
oenocyte-less - male (HC coated)	Trial 3	212.5 (19.5)	54.7 (5.2)	1329.3 (122.6)	na	1596.4 (147.1)	3

Journal of Experimental Biology • Supplementary information
